# 2-Aminoethylphosphonate Utilization by the Cold-Adapted *Geomyces pannorum* P11 Strain

**DOI:** 10.1007/s00284-013-0485-4

**Published:** 2013-10-27

**Authors:** Magdalena Klimek-Ochab, Artur Mucha, Ewa Żymańczyk-Duda

**Affiliations:** Department of Bioorganic Chemistry, Faculty of Chemistry, Wroclaw University of Technology, Wybrzeże Wyspiańskiego 27, 50-370 Wrocław, Poland

## Abstract

Cold-adapted strain of *Geomyces pannorum* P11 was found to mineralize of phosphorus–carbon bond-containing compound—2-aminoethylphosphonic acid (2-AEP, ciliatine). The biodegradation process proceeded in the phosphate-independent manner. Ciliatine-metabolizing enzymes' activity was detectable in cell-free extracts prepared from psychrophilic *G. pannorum* pregrown on 4 mM 2-AEP. Phosphonoacetaldehyde hydrolase (phosphonatase) activity in a partially purified extract was demonstrated at 10 °C.

## Introduction

Phosphorus is an essential element for many biomolecules and plays an important role in various biological processes. Phosphonates are a class of organophosphorus molecules characterized by a stable carbon–phosphorus (C–P) bond, which resists chemical, thermal, and photolytic degradations [22]. 2-Aminoethylphosphonate (ciliatine) and its *N*-alkylated derivatives are the most abundant and ubiquitous of naturally occurring phosphonates [2, 13]. The ciliatine-based structural fragments are typically found as conjugates of glycans [1], lipids [15], and proteins [12], which in turn perform essential biochemical functions in specialized lower organisms. Because of the natural origin of 2-aminoethylphosphonic acid (2-AEP), microorganisms have acquired a unique pathway its degradation to usable forms of carbon (C), phosphorus (P), and nitrogen (N) [6, 9, 17–19, 24, 28].

The biodegradation pathway of ciliatine molecule is described as a two-step process. The first reaction reported as transamination is carried out by 2-AEP transaminase and leads to the formation of phosphonoacetaldehyde and corresponding amino acid. This enzyme [EC 2.6.1.37] has been isolated from *Pseudomonas aeruginosa* [9] and *Salmonella typhimurium* [6, 16]. Transaminase requires pyridoxalphosphate as cofactor and is specific for pyruvate as the amino group acceptor, while 2-AEP acts as amino group donor. The next step includes hydrolytic cleavage of the C–P bond within the phosphonoacetaldehyde molecule and results in formation of inorganic phosphate and acetate. This reaction is carried out by an enzyme phosphonoacetaldehyde hydrolase (phosphonatase) [EC 3.11.1.1] that has been purified from *Bacillus cereus* [24, 30]. Both enzymes involved in ciliatine degradation are synthesized in microbial cells and are inhibited by the presence of inorganic phosphate. The literature data concerning the phosphate starvation-independent ciliatine biodegradation are scarce [18, 28].

Currently, the biodegradation of ciliatine was mainly studied using mesophilic microorganisms, whereas there is a limited knowledge of the ability of extremophilic microbes to utilize this compound. Indeed, the only documented study reported successful application of a thermophilic strain of *Geobacillus* *caldoxylosilyticus* T20 for utilization of a number of structurally variable organophosphonates as a sole P source for growth at 60 °C [23].

So far, degradation pathways have not been investigated in cold-adapted organisms. In this paper, for the first time, we report bioconversion of 2-AEP, which is driven at 10 °C, by a psychrophilic fungal strain of genus *Geomyces.* Importantly, this process appeared to be independent on the presence of phosphate in the growth medium. Ciliatine is metabolized for fungal cell purposes, and P–C bond cleavage enzymatic activity of crude extract has been demonstrated.

## Materials and Methods

### Chemicals

All chemicals were of the highest purity available and were procured from Sigma-Aldrich (Poland), POCh (Poland), except for the phosphonoacetaldehyde dilithium salt (phosphonatase substrate). This compound was synthesized according to procedure described below.

### Microorganism

The psychrophilic fungal strain named P11 was a generous gift from Prof. Marianna Turkiewicz (Institute of Technical Biochemistry, Technical University of Lodz). The microorganism was identified in DSMZ Laboratory (Germany). Sequencing of the rDNA ITS fragment revealed a 99 % similarity to *Geomyces pannorum* NRBC 31776 and 98 % to *G. pannorum* ATCC 11501. Accordingly, the strain of P11 was identified as *G.* *pannorum* (Link) Sigler and J. W. Carmich (MB 314399).

Fungus was routinely maintained on Czapek–Dox agar, which provided profuse sporulation suitable for inoculum collection.

### Growth Conditions

Degradation experiments were carried out using modified Czapek liquid medium, which consisted of: 30 g l^−1^ sucrose, 0.5 g l^−1^ MgSO_4_·7H_2_O, 0.5 g l^−1^ KCl, 2.64 g l^−1^ NaNO_3_, 3.0, 0.01 g l^−1^ FeSO_4_·7H_2_O, and 0.5 g l^−1^ KH_2_PO_4_, at pH 7.2. Depending on the outcome, the C or phosphate or N source was omitted and replaced by filter-sterilized 2-AEP; in the latter case, the medium was buffered with 5 mM HEPES–KOH, pH 7.2. Thus C source was supplied at final concentration of 8 mM, N source (as well N and P) at a final concentration of 4 mM, and P at final concentration of 2 mM. The generation time of psychrophilic fungi is very long and to obtain enough biomass for enzymatic tests, they were cultivated both stationary and on rotary shaker. Cultures were grown either in 15-cm Petri dishes containing 50 ml of liquid medium or at 135 rpm in 250-ml Erlenmeyer flasks containing 100 ml of the medium. Cultivation medium was inoculated with a spore suspension in 0.05 % Triton X-100 to a density of 10,000 spores ml^−1^ and incubated at 10 °C. Mycelium was harvested by vacuum filtration onto filter paper. Fungal growth was evaluated by dry mass determination: filters with fungal cells were dried for over 48 h in an oven at 70 °C and then weighted.

### Dilithium 2-Oxoethylphosphonate (Phosphonoacetaldehyde Dilithium Salt) Synthesis

2-Acetoxy-2-chlorophosphonyl dichloride was prepared by passing gaseous sulfur dioxide (generated from Na_2_S_2_O_5_ and sulfuric acid) through suspension of the product of the reaction between vinyl acetate and phosphorus pentachloride, following the procedure of Lutsenko and Kirilov [21]. Yield 50 %, bp 135–138 °C, 12 mmHg (lit. bp 99–100 °C, 1.5 mmHg [21]; 83 °C, 0.3 mmHg [10]. ^1^H NMR (300 MHz, CDCl_3_) *δ* 2.18 (s, 3H, CH_3_), 3.42 (ABXY system, *J*_AB_ = 15.6 Hz, *J*_HA_ = 8.9 Hz, *J*_HB_ = 3.7 Hz, *J*_PA_ = 14.0 Hz, *J*_PB_ ≈ 15.6 Hz, CH_2_), 6.81 (ddd, *J*_P_ ≈ *J*_HA_ = 8.9 Hz, *J*_HB_ = 3.7 Hz, CH). ^31^P NMR (300 MHz, CDCl_3_) *δ* 36.06. The dichloride was dissolved in THF and hydrolyzed with 3 equiv. of water to phosphonoacetaldehyde according to the method described by Isbell et al. [14]. After careful removal of the volatile components under reduced pressure and drying in a desiccator, the oily residue was redissolved in water and treated with LiOH to pH 9 [20]. The final product was precipitated quantitatively with addition of a high volume of acetone, filtered, washed thoroughly with acetone, and dried in a desiccator. ^31^P NMR (300 MHz, D_2_O) indicated two signal corresponding to the aldehyde (*δ* 9.78) and its *gem*-diolate hydrate (*δ* 14.52) in a 2:5 integration ratio.

### Analytical Methods

Ciliatine residual concentration in medium supernatants was determined using colorimetric method, based on Ninhydrin Reagent (2 % solution; Sigma) according to the producer recommendation. This method may be used for the determination of the concentration of primary and secondary amines and amino acid in solutions. The effective range of 2-AEP that could be measured by means of this method, was from 0 to 0.3 mM. Ammonium sulfate was used as a standard to create a standard curve.

Inorganic phosphate was quantified colorimetrically by means of the green malachite acid dye assay [10]. This method enables the measurement of inorganic free phosphate in aqueous solutions. It is based on the formation of specific complex between malachite green molybdate and free orthophosphate that absorbs at 620–640 nm. The presence of phosphonate or other bound P does not interfere with the final results. Potassium monophosphate was used as a standard to create a standard curve, performed for each freshly prepared stock of reagent mixture.

Protein concentration was measured by the method of Bradford [4], using bovine serum albumin solutions as the standard. ^31^P NMR analysis of culture supernatants was performed to confirm the biodegradation of substrate. They were done after centrifugation of cells and culture broth evaporation to dryness. The obtained residue was dissolved in 0.5 ml of D_2_O and analyzed using DRX Bruker apparatus (Karlsruhe, Germany) operating at 121.50 MHz. All determinations and treatments were carried out at least in triplicate; reported values are mean ± SD over replicates.

### Preparation of Cell-Free Extract and Hydrolase Activity Assay

Cultures were grown to mid-exponential phase, then harvested by centrifugation, and the mycelium was washed twice with ice-cold 50 mM Tris–HCl buffer, pH 8, ground in a mortar with quartz sand, and subsequently disrupted in a Cole Parmer Torbeo 36800 600-W sonicator with four cycles of 30 s sonication and 2 min cooling. Cell debris was removed by centrifugation at 20,000×*g* for 15 min at 4 °C. The supernatant was dialyzed against 50 mM Tris–HCl buffer, pH 8.0 overnight, and loaded onto a DEAE-Sephacel column equilibrated with the same buffer. Retained proteins were eluted with a minimal amount of buffer containing 250 mM NaCl, and the resulting extract was assayed toward C–P bond cleavage activity. The above steps of extract preparation, starting from the dialysis, were performed at a cold room (10 °C).

### Cell-Free Extract Activity Assays

C–P bond cleavage activity in fungal cell-free extract prepared from 2-AEP utilizing *Geomyces* strain was assayed in a coupled transamination-dephosphonylation reaction by a modified Cook et al. method [8]. The reaction mixture (100 μl) contained 50 mM Tris–HCl buffer, pH 8, 5 mM MgCl_2_, 1 mM pyridoxal-5-phosphate, 0.5 mM DTT, 0.5 mM EDTA, and 5 mM 2-AEP. Sodium pyruvate, α-ketoglutaric acid, and oxalacetic acid were introduced into the reaction mixture at 10 mM concentrations individually or in their combinations and were tested as possible amino functionality acceptors. The reaction was carried out at 10 °C. After an appropriate incubation period (up to 60 min), the inorganic phosphate released was quantified colorimetrically. The hydrolase activity was expressed as the amount of phosphate released s^−1^ (mg protein)^−1^.

## Results and Discussion

Psychrophilic microorganisms and products of their metabolism have potential applications in a broad range of industrial, agricultural and medical processes [5]. In order to grow in low-temperature environments, all cellular components must adapt to cold. Cold active enzymes offer novel-varied opportunities for biotechnological exploitation based on their high catalytic activity at low temperature, decreased thermostability, and unusual specificities [26]. Despite extensive research focused mainly on a biochemical characterization and taxonomic description of psychrophilic microorganisms, our understanding of the participation of psychrophilic bacteria and fungi in biodegradation processes remains limited. The current study is the first, which reports the ability of cold-adapted fungal strain, *G. pannorum* P11 to metabolize a natural organophosphonate—ciliatine.

**Fig. 1:**
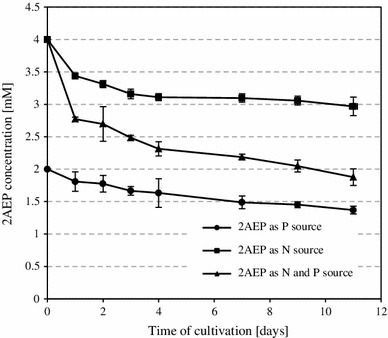
Fungal utilization of 2-AEP used as a source of: phosphorous, phosphorus and nitrogen, or only nitrogen in the cultivation medium. Data are mean ± SD of three replications

Fungal strain of P11 was found to grow in modified Czapek liquid medium, containing either 2 mM phosphonate as the only source of phosphate, or 4 mM 2-AEP as a source of N or 4 mM 2-AEP as a source of both P and N. The analysis of culture supernatants clearly demonstrated that phosphonate was removed to some extent from every culture variations (Fig. 1). Complete removal of ciliatine, however, was not achieved and the fungal degradative efficiency observed at the 11th day of experiment was: 22 %, when ciliatine served as the P source, 28 % when 2-AEP was the only N source and 53 % if phosphonate was used as both P and N source. Uninoculated controls showed no decomposition of the substrate. Biomass yields obtained in each of these experiments were comparable or even higher to those obtained upon cultivation of the fungal strain in complete medium, containing inorganic phosphate and ammonium ions (data not shown). Thus, when supplied as both P and N sources, ciliatine was gradually removed from the culture broth, with accompanying release of inorganic phosphate into the medium, starting 3–4 days after inoculation. However, the final concentration of Pi in culture broth did not exceed 0.15 mM (data not shown).

The degradative activity towards ciliatine was also studied in *G. pannorum* P11 cultivated under stress conditions. Fungal biomass obtained from culture grown in non-modified Czapek medium was starved for 24 h in water and then transferred into the modified medium supplemented in 4 mM of 2-AEP applied as a source of both P and N. Fungus was cultivated for 11 days and the progress of substrate degradation was followed by measuring of inorganic phosphate liberation into the culture medium and by the reduction of substrate concentration (Table 1). This procedure allowed to obtain high amount of fungal biomass with active enzymes involved in 2-AEP degradation and allowed performing a number of enzymatic assays in very short time. The analysis of culture supernatants clearly demonstrated that ciliatine was removed from the culture medium concomitant with inorganic phosphate release to the medium. Our findings indicate that both actions: the uptake and the utilization of 2-AEP are independent on the phosphate status of the cell and are induced by the substrate present in the environment.

**Table 1:** The utilization of 2-AEP by *Geomyces pannorum* P11 under stress condition and the level of phosphate liberated into the medium

Time of cultivation (days)	PA residual level (mM)	Pi liberated (mM)
0	4	0.069 ± 0.009
1	3.62 ± 0.003	0.068 ± 0.004
2	3.68 ± 0.005	0.11 ± 0.03
4	3.03 ± 0.06	0.17 ± 0.009
5	2.78 ± 0.05	0.20 ± 0.009
8	2.24 ± 0.04	0.25 ± 0.001
11	1.69 ± 0.04	0.30 ± 0.006

Biomass obtained from cultures grown for 7 days in non-modified Czapek medium, was transferred, after 24 h starvation period into the fresh Czapek-no Pi medium with 4 mM PA as N and P sources and concentration of PA and Pi in this culture broth were measured. Data are means ± of three independent replications

The examined cold-adapted fungal strain can possess a Pi-deregulated cellular transport system. Studies on the fungal transport of anions—phosphate, sulfate, nitrate, and organic acids are scarce. Transport processes in fungal cells have been studied mainly in mesophilic molds, whereas systems that function in psychrophilic fungi are poorly investigated. Two transport systems defined as high affinity and low affinity have been reported for *Aspergillus nidulans* and *Neurospora crassa* [11]. Both systems were constitutive and not dependent on the phosphate concentration in the growth medium. However, the kinetic parameters of these systems varied with the cells growth conditions. Aminophosphonate substrates can be also uptaken by fungal cells via amino acids transport system, based on the permease activity. Such transport mechanisms were proposed in the studies of organophosphonate metabolism in *Candida maltosa* [3] and *Kluyveromyces fragilis* [27]. Accordingly to those suggestions, yeasts possessed a general amino acid permease, which allowed transporting the aminoalkylphosphonates such phosphonoalanine and 4-aminobutylphosphonate, respectively, into the cell.

**Fig. 2:**
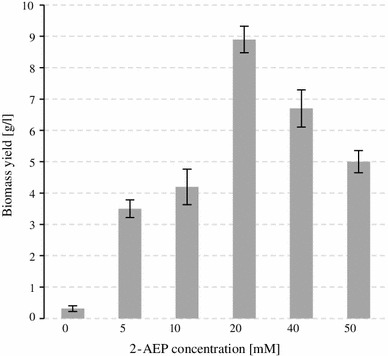
The influence of increasing concentrations of 2-AEP on fungal growth. Data are mean ± SD of three replications

The influence of increasing concentration of 2-AEP on *Geomyces pannorum* P11 growth was also studied. The solutions of the substrate supplied as the P source up to 20 mM stimulated the fungal growth, and lower biomass yields were observed when the ciliatine level exceeded this value (Fig. 2). Such an observation is in a good agreement with those obtained previously for *Penicillium purpurogenum*, which is able to mineralize the ciliatine [18].

For the first time, in vitro activity of fungal crude extract against a phosphonate molecule was demonstrated for cold-adapted microbe, what is a direct evidence for the presence of enzymes responsible for cleavage of the C–P bond. Cell-free extracts prepared from mycelium grown on 2-AEP as the source of N and P exhibited detectable 2-AEP transaminase and phosphonatase activities against this substrate, if assayed at 10 °C. Specific activities under saturating substrate concentration (10 mM) ranged from 0.56 to 0.80 nmol of Pi released s^−1^ (mg protein)^−1^. These results are comparable to those obtained for *P. purpurogenum* strain [18]. An increase of temperature of sample incubation up to 20 °C caused the decrease of enzymatic activity of fungal crude extract to 0.10 nmol of Pi (mg protein)^−1 ^s^−1^. The level of the crude extract activity towards ciliatine, measured with the addition of the only one amino functionality (becoming from 2-AEP) acceptor available in reaction mixture, varied depending on the chosen compound (Table 2).

**Table 2:** The level of *Geomyces* cell-free extract specific activity towards ciliatine (10 mM) measured in the presence of different amino group acceptors

Amino group acceptors	Specific activity [nmol of Pi (mg protein)^−1 ^s^−1^]
Sodium pyruvate	0.32
α-Ketoglutarate	0.13
Oxalacetate	0.28
All	0.56

The highest level of the C–P bond cleavage activity was obtained in the crude extract, when all acceptors for initial transamination of ciliatine were present in the samples. Surprisingly, the lowest level of discussed enzymatic activity was observed when α-ketoglutarate was added as the amino group acceptor. It was surprising because this keto acid is a common, physiological amino group acceptor allowing N fixing in eukaryotic cells. Considering this, our observation is unusual and probably it illustrates one of metabolic differences that distinguish cold-adapted organisms from mesophilic ones.

The obtained results are distinct from both those reported for bacterial strain metabolizing ciliatine and for fungi able to growth on media containing phosphonates as source of N and P. Bacterial transaminase from various sources showed a very high enzymatic specificity since only ciliatine and pyruvate are amino functionality donor and acceptor, respectively [9, 16, 28]. In the case of *Geomyces* P11 cell-free extracts, all possible acceptors, namely, pyruvate, α-ketoglutarate, and oxaloacetate are promoting the reaction. Moreover application of the mixture of acceptors resulted in the highest level of transaminase activity. This finding is similar to fungal transaminase from *P. purpurogenum*, which also used every of these amino residue acceptors, with the α-ketoglutarate yielding the highest enzymatic activity. The level of enzymatic activity of *Penicillium* crude extract, obtained when all acceptors were available in the samples was very low, suggesting possible competition between them. Thus a strong difference between biochemically examined fungal strains is clearly seen.

Additionally, cell-free extracts prepared from mycelium grown on 2-AEP as the only source of N and P contained hydrolytic activity against the phosphonoacetaldehyde (10 mM) and the activity of second enzyme (phosphonoacetaldehyde hydrolase) involved in ciliatine degradation could also be demonstrated. Phosphonatase activities up to 32.79 nmol of Pi released s^−1^ (mg protein)^−1^ were detected.

After storage of fungal crude extract in 4 °C for 24 h, significant changes in enzymes activities occurred and no enzymatic activity towards 2-AEP was detected. Surprisingly, these crude extracts still exhibited a very high level of enzymatic activity towards phosphonoacetaldehyde (10 mM) up to 30.65 nmol of Pi (mg protein)^−1 ^s^−1^ what indicted that two crucial enzymes involved in the phosphonate degradation process, exhibited various sensitivity to the temperature values. The transaminase seemed to be a very temperature-sensitive enzyme, whereas the thermal stability of phosphonatase is noticed.

Deeper characterization of the proteins involved in biodegradation of 2-AEP, leading to extend the information about enzymes, with respect to properties and regulative mechanisms, thus this work is currently in progress starting from purification of the enzymes to electrophoretic homogeneity.

## Conclusions

Regardless of the physiological significance of the described project, the study of ciliatine catabolism in psychrophilic fungi may have interesting implications in the development of new bioremediation strategies and expand our knowledge of biodiversity of microbes able to mineralize P–C bond consisting compounds.

Moreover, ciliatine is an important precursor, used in the biosynthesis of cellular metabolites performing essential biochemical functions in lower organisms. In pathogens, 2-AEP conjugates are used for host infection and persistence [7, 25, 29]. This phosphonate compound is present as a component of complex carbohydrates on the surface membrane of many trypanosomatids (*T. cruzi*, the etiological agent of the Chagas disease). In each parasite stage, molecules (including 2-AEP), belonging to the membrane surface are the ones most probably involved in interaction with the host [25, 29]. Therefore, the enzymes involved in 2-AEP metabolism are targets for the design of antimicrobial and antiparasite inhibitors.
